# Hearing function of gold miners with and without a history of tuberculosis treatment: a retrospective data review^☆^

**DOI:** 10.1016/j.bjorl.2018.12.003

**Published:** 2019-01-23

**Authors:** Katijah Khoza-Shangase

**Affiliations:** University of the Witwatersrand, School of Human and Community Development, Department of Speech Pathology and Audiology, Johannesburg, South Africa

**Keywords:** Mines, Monitoring, Noise, Ototoxicity, Tuberculosis, Minas, Monitoramento, Barulho, Ototoxicidade, Tuberculose

## Abstract

**Introduction:**

South Africa has a high prevalence of co-existing tuberculosis and HIV. As ototoxicity linked to the treatments for these conditions occurs with concomitant exposure to other ear toxins such as hazardous noise exposure, it is important to investigate the combination impact of these toxins. Limited published evidence exists on the co-occurrence of these conditions within this population.

**Objectives:**

The objective of this study was to compare the hearing function of gold miners with (treatment group) and without (non-treatment group) the history of tuberculosis treatment, in order to determine which group had increased risk of noise induced hearing loss. Furthermore, possible influence of age and HIV in these two groups was examined.

**Methods:**

A retrospective record review of 102 miners’ audiological records, divided into two groups, was conducted, with data analyzed both qualitatively and quantitatively.

**Results:**

Findings suggest that gold miners with a history of tuberculosis treatment have worse hearing thresholds in the high frequencies when compared to those without this history; with evidence of a noise induced hearing loss notch at 6000 Hz in both groups. Pearson's correlations showed values between 0 and 0.3 (0 and −0.3) which are indicative of a weak positive (negative) correlation between HIV and hearing loss, as well as between hearing loss and age in this population.

**Conclusions:**

Current findings highlight the importance of strategic hearing conservation programs, including ototoxicity monitoring, and the possible use of oto-protective/chemo-protective agents in this population.

## Resumo

**Introdução:**

A África do Sul apresenta uma alta prevalência de coinfecção de tuberculose e HIV. Como a ototoxicidade associada aos tratamentos para essas condições é observada na exposição concomitante a outros agentes ototóxicos, como a exposição a ruídos perigosos, é importante investigar o impacto da combinação desses agentes. São poucas as evidências publicadas sobre a co-ocorrência dessas condições nessa população.

**Objetivo:**

Comparar a função auditiva de garimpeiros com (grupo tratamento) e sem (grupo sem tratamento) história de tratamento de tuberculose, a fim de determinar que grupo apresentava maior risco de perda auditiva induzida por ruído. Além disso, avaliou-se a possível influência da idade e do HIV nesses dois grupos.

**Método:**

Os registros audiológicos de 102 garimpeiros, divididos em dois grupos, foram revisados de forma retrospectiva; os dados foram qualitativa e quantitativamente analisados.

**Resultados:**

Os achados indicam os garimpeiros com histórico de tratamento de tuberculose apresentam piores limiares auditivos nas altas frequências quando comparados àqueles sem esse histórico; em ambos os grupos, observou-se perda auditiva induzida por ruído com entalhe audiométrico a 6.000 Hz. As correlações de Pearson mostraram valores entre 0 e 0,3 (0 e -0,3), que são indicativos de uma fraca correlação positiva (negativa) entre o HIV e a perda auditiva, bem como entre a perda auditiva e a idade nessa população.

**Conclusões:**

Os resultados atuais destacam a importância de programas estratégicos de conservação auditiva, inclusive monitoramento de ototoxicidade, e o possível uso de agentes oto-/quimioprotetores nessa população.

## Introduction

Numerous health challenges plague the gold mining industry within South Africa, including occupational noise induced hearing loss (NIHL) as well as occupational lung diseases such as tuberculosis (TB).^1^ Within the gold mining industry, health concerns such as NIHL have been noted to remain static, even with the attempt of employing techniques aimed at silencing of equipment.^1^ Moreover, TB within the South African context has been shown to grow dramatically, and has been correlated with the increased prevalence of Human Immunodeficiency Virus (HIV).^2^ Although the TB and HIV epidemics are seen throughout Southern Africa, according to the AngloGold Ashanti West Wits country report,^1^ it was estimated that approximately 85% of their employees were diagnosed with TB as well as HIV. The implications of the co-existence of these conditions with established relationships to hearing function, within a chronic noise-exposure environment require investigating and quantification. Hence the importance of the current study.

The mining industry represents 8% of South Africa's Gross Domestic Product (GDP), and is arguably one of the country's most important industries.^1^, ^3^, ^4^ As much as there are employment opportunities within the gold mining industry, enough evidence exists to support the fact that there is a human cost linked to the mining of gold. Studies have been conducted that documented health problems of miners, which include decreased life expectancy as well as an increased prevalence of occupational pulmonary TB and NIHL.^1^, ^5^, ^6^, ^7^, ^8^

In 2012, AngloGold Ashanti reported that there were 446 new cases of TB amongst their employees.^1^ In this particular report, the company acknowledged that an increase in HIV/AIDS infections brings an interdependency of the increase in TB cases in the South African context. These challenges bring with them the economic impact of illness and pathology.^9^ Karim et al.^10^ argues that South Africa is experiencing the world's worst HIV and TB pandemic; hence the need for careful and clear understanding of the impact of TB treatment in the presentation of NIHL within the South African context.

TB within the South African gold mining industry can be seen as a major health concern. This concern highlights the need to describe hearing functions of gold miners with and without the history of TB treatment, hence the current study.

Specific objectives of this research were:

(1) To describe and compare the hearing function of a group of gold miners with a history of TB treatment (treatment group) to that of a group without the history (non-treatment group) – both groups exposed to noise.
(2) To establish a possible relationship that age and HIV might have on hearing function of these two groups of miners.

## Methods

### Research design

A quantitative cross-sectional retrospective record review design was adopted, through data obtained from 102 (204 ears) participant medical files where hearing function of noise exposed miners with a history of being previously re-treated for TB was compared to those with no history of TB treatment, to establish who had increased risk for noise induced hearing loss. Purposive sampling was used to select the participant files who met the inclusion criteria for the study. However, because one of the aims of the study was to attempt to establish the possible impact that history of TB treatment may have on hearing function in miners, it was crucial to apply a set of participant inclusion criteria. Given the fact that a paucity of published research on this aspect of occupational noise induced hearing loss in South Africa exists, the researcher believed that it was crucial to have oversight over variables which could confound the results of the study (for example, HIV and its treatments, syphilis, chronic exposure to mercury, chronic smoking, and so forth). Hence the inclusion criteria listed below.

Each group of gold miners comprised of 51 participant files (102 ears each), and participants were matched for age, gender, and years of exposure to occupational noise.

The participant inclusion criteria were:

Positive/negative history of past TB infection and past TB re-treatment for the treatment and non-treatment groups respectively. The treatment group only included participants’ audiograms post the re-treatment phase but matched with years of exposure and noise exposure in the non-treatment group.

Age between 18 and 56 years.

Available serial audiologic monitoring records.

Participants with other possible causes of hearing loss such as middle ear pathology, history of meningitis, syphilis, use of chemotherapeutic agents, recreational noise exposure, etc., were excluded.

### Testing protocol

Prior to commencement of the study, permission to conduct the research project was secured from the University's Medical Human Research Ethics Committee (Protocol n° M140331). Moreover, the study was performed in accordance with the ethical standards laid down in the 2012–2013 World Medical Association's Declaration of Helsinki. Permission to conduct the study at the mine was obtained from mine management and from the relevant heads of departments. Once permission was obtained and ethical clearance secured, the researcher conducted detailed record reviews at the research site.

### Data analysis and statistical procedures

In order to ensure research reliability, controls were exercised pertaining to participant variables, parameters pertaining to the records reviewed and data analysis procedures employed. Over and above conducting record reviews, utilizing an independent rater during data analysis, a pilot study was also conducted to ensure reliability and validity of the data capturing tool.

To analyze data, the mean and standard deviation were calculated for each group. The mean scores were calculated for age, each PT (pure tone) frequency in each ear, as well as the PLH (percentage loss of hearing). Then, a two-sample, one-tail *t*-test was done in order to identify the difference in hearing status between the two groups. Next, the two-sample, one-tail *t*-test was performed to determine if HIV had an impact on hearing status. Lastly, Pearson's correlations were done in order to identify possible relationships between age and hearing loss, as well as between HIV and hearing loss. Correlation coefficients are expressed as values between +1 and −1; with +1 indicating a perfect positive correlation, while 0 indicates no correlation. Values between 0 and 0.3 (0 and −0.3) indicate a weak positive (negative) correlation; while values between 0.3 and 0.7 (−0.3 and −0.7) indicate a moderate positive (negative) correlation. Values between 0.7 and 1 (−0.7 and −1) indicate a strong positive (negative) correlation.

## Results

The sample (Table 1) contained gold miners with (treatment group) and without (non-treatment group) a history of TB treatment from the chosen gold mine. The study contained 102 participant medical files (204 ears). Participants’ mean age ranged from 41 to 48 yrs (mean = 43 yrs), with the average length of employment at the mine (length of exposure to noise) being 11.5 yrs (range 5.6–13.4 yrs). For the treatment group, the average length of time of the audiogram reviews post TB treatment was 1.5 yrs (range 9 months to 1.4 yrs). All participants in the treatment group had a history of streptomycin use with records of capriomycin, amikacin or kanamycin aminoglycosides use. Of the treatment group, 76% also presented with HIV (as opposed to 9% in the non-treatment group) with inconsistently documented history of Antiretroviral (ARV) treatment as well as CD4+ or viral load data. For those on ARV treatment, their treatment was in line with the adult antiretroviral therapy guidelines.

**Table 1:** Summary of demographic and medical details of participants.

Factor	Non-treatment group	Treatment group
Participants mean age (yrs)	41.3	45.03
Participant HIV status	NEG (negative): 91%	NEG: 24%
	POS (positive): 9%	POS: 76%

### Description and comparison of the hearing function in both groups

**Figure 1:**
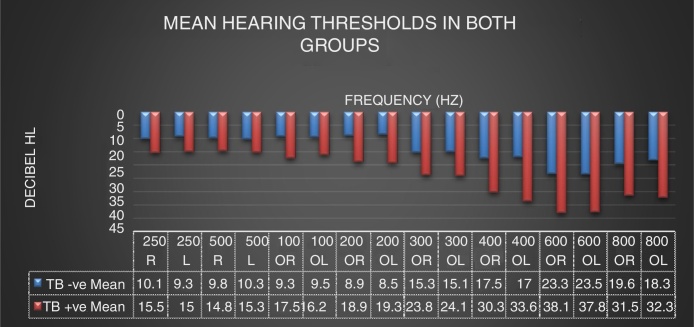
Mean hearing thresholds in both groups (*n* = 204 ears).

On analysis of the mean hearing thresholds on audiograms (Fig. 1), results revealed hearing thresholds below 25 dB (within normal range of hearing) for all frequencies tested in the non-treatment group. Although still within normal limits, a bilateral notch at 6000 Hz was found in the non-treatment group. Although the dip is present in both groups, the mean pure tone thresholds are worse in the treatment group than in the non-treatment group – in both ears. Hearing thresholds in the treatment group were worse than 25 dB in high frequencies (4000, 6000 and 8000 Hz) where thresholds were worse than 30 dB HL. These findings indicate mild high frequency sensorineural hearing loss in the treatment group.

The thresholds in the low and mid frequency range (500, 1000, 2000 and 3000 Hz) are better than 25 dB (within normal limits) in both groups.

Results from the two sample one-tailed *t*-test used to determine if there was a difference in the hearing status between the two groups indicated that most of the cases rejected H_0_, implying that there was a statistically significant difference in hearing ability between both groups at all frequencies tested except at 250 Hz bilaterally. This finding was for all frequencies tested bilaterally (except 250 Hz). When both ears scores were combined, statistically significant differences were found at all frequencies, except at 250 Hz (significance was 5%, indicating that there was a confidence level of 95%).

### Possible relationship between age, HIV and hearing loss

Results from the two-sample one-tailed *t*-test used to identify if HIV had an impact on hearing status in the current sample indicated that H_0_ was not rejected at any of the frequencies included, implying that HIV did not have an impact on the hearing status of gold miners in this population.

Pearson's correlations were done in order to identify possible relationship between age and hearing loss, as well as HIV and hearing loss in both groups. Values between 0 and 0.3 (0 and −0.3) were obtained; and these are indicative of a weak positive (negative) correlation (as depicted in Table 2, Table 3); implying that these factors were not exacerbating factors on this sample's hearing status.

**Table 2:** Pearson's correlation figures for age and hearing loss.

Correlation figures for non-treatment group – age and hearing loss														
	PT 500		PT 1K		PT 2K		PT 3K		PT 4K		PT 6K		PT 8K	
	Right	Left	Right	Left	Right	Left	Right	Left	Right	Left	Right	Left	Right	Left
*r*	−0.3	−0.1	0.28	−0.1	0.3	0.1	0.4	0.2	0.26	0.14	0.24	0.3	0.06	0.3

Correlation figures for treatment group – age and hearing loss														
	PT 500		PT 1K		PT 2K		PT 3K		PT 4K		PT 6K		PT 8K	
	Right	Left	Right	Left	Right	Left	Right	Left	Right	Left	Right	Left	Right	Left
*r*	−0.3	−0.2	−0.06	−0.14	0.2	0.1	0.13	0.1	0.3	0.24	0.3	0.14	0.08	0.14

**Table 3:** Pearson's correlation figures – participants HIV.

Participants HIV positive														
	PT 500		PT 1K		PT 2K		PT 3K		PT 4K		PT 6K		PT 8K	
	Right	Left	Right	Left	Right	Left	Right	Left	Right	Left	Right	Left	Right	Left
*r*	−0.2	−0.1	−0.02	−0.2	0.1	−0.14	0.12	−0.03	0.3	0.2	0.1	0.1	−0.12	−0.14

## Discussion

The prevalence of NIHL in the gold mining industry still persists despite South African legislation that requires mines to implement hearing conservation programs. It is therefore important to note that intrinsic and environmental influences can add to the effects of occupational noise exposure, as seen in the current study with history of TB treatment.

Current findings indicate, firstly, that all the noise-exposed gold miners in the group without the history of TB treatment had hearing function within normal limits. However, there was a clear notch at 6000 Hz, where the hearing threshold level was worse in both groups. This notch at 6 kHz is consistent with the documented configuration of a noise-induced hearing loss dip at this frequency. According to Humes et al.,^11^ chronic noise exposure may be indicated by a notch that is generally situated at 3000, 4000 or 6000 Hz. The variation of location of the notch is purely due to the type of noise to which individuals are exposed.^12^ Excessive noise exposure is considered a global occupational health hazard and enough evidence exists that shows a strong correlation between occupational noise and hearing loss which typically starts with this notch.

Although the notch is present in both groups, the pure tone threshold mean is significantly worse in the treatment group than in the non-treatment group. This is the first evidence in the current sample indicating that gold miners with a history of TB treatment are at increased risk of NIHL as indicated by the worse notch at 6000 Hz when compared to the group without history of TB treatment. Secondly, current findings clearly demonstrate a high frequency sensorineural hearing loss in the treatment group, which was absent in the non-treatment group. This is the second evidence linking TB to hearing loss in this study. As all conditions and variables were fairly equal in both groups, except for the TB treatment variable, it can be argued that current findings can be attributable to the nature of ototoxic medication which is part of TB treatment regimen. Treatment of TB, via medication such as streptomycin, has been documented to cause ototoxic hearing loss in many individuals with certain risk factors. These risk factors include the dosage and duration of TB medication, nutrition, psychological state, age, renal function, as well as pre-existing hearing status.^13^ Streptomycin is a highly ototoxic drug and may have implications on the cochlea through deterioration of the outer hair cells, especially affecting the higher frequencies as in the current findings – another confirmation of the causal relationship between the history of TB treatment and hearing loss in the treatment group.

According to Roland and Rutka,^14^ ototoxic medication such as that in TB medication, affects the basal turn of the cochlear causing high frequency hearing loss and/or tinnitus. These authors also state that lower and mid-frequencies may also be involved, depending on the length of exposure to ototoxic medication, thus causing an overall hearing loss.^14^ Current findings are therefore consistent with this evidence.

Dalebout^15^ argues that for theoretical and pathophysiological reasons, hearing loss caused by noise exposure and/or ototoxic medication is known to affect both ears equally and symmetrically, therefore no difference in hearing function was expected between the right and left ears in the current study. Current findings where Ho was not rejected (there was no significant difference in hearing between the left and right ears) therefore, supported Dalebout's^15^ theory of symmetrical effects of these ear toxins.

The fact that evidence of NIHL (notch at 6000 Hz) was seen in both groups with, however, additional high frequency hearing loss in the treatment group seems to confirm that exposure to TB treatment worsens hearing function in this noise-exposed population. This finding supports the documented evidence that TB treatments have a direct effect on an individual's hearing; and that concomitant exposure to ototoxic medications and noise imposes synergistic effects on the ear; thereby compounding the presenting hearing symptom.^16^

According to Valente et al.,^17^ HIV is documented to cause a sensorineural hearing loss in approximately 49% of infected patients. Local studies^18^, ^19^, ^20^, ^21^, ^22^, ^23^ have indicated a link between HIV/AIDS and hearing function in both adults and children with the causal links being direct effects, secondary effects (opportunistic infections) or iatrogenic (ototoxicity of treatments). Despite this evidence of an established relationship between HIV/AIDS and hearing loss, the current study evidence did not show that HIV had any significant impact on the hearing status of participants in the study. This could be due to the current study's small sample size effects. The fact that the retrospective record review nature of the study presented with limitations in the data obtained, such as CD4+ counts, viral loads, and ARV treatments data; prevents the current study from making any conclusive findings regarding this HIV variable. This therefore raises an implication for future studies.

The lack of correlation between age and hearing loss in both groups as a finding is not surprising. In the current study, the average age of gold miners without a history of TB treatment was 40.2 yrs and 45.4 yrs for those with a history of treatment. The natural deterioration of hearing with age becomes a factor during approximately the 5^th^ and 6^th^ decade of life.^24^ It must be noted that individual hearing may vary and this statement may not be true for all individuals. In non-noise exposed populations, this age of presbycusis explanation is understandable. However, in noise-exposure, current findings contradict some evidence which suggests age-noise interaction that exacerbates age-related hearing loss.^25^ Current findings, nonetheless, add strength to the link between the hearing loss found in the treatment group in this study to TB treatment rather than presbycusis.

## Conclusion

Current findings underscore the need for audiological involvement in the monitoring of hearing function in miners in this population. This need appears to be more pressing in miners with a history of TB treatment since these miners seem to present with worse hearing than those in the non-treatment group. Furthermore, the fact that HIV raises individual susceptibility to TB, as confirmed in the current study where 70% of participants with a history of TB treatment had HIV, means increased demand for audiology services in the South African noise exposed mining population who have TB and are HIV positive.

Findings from this study are based on a relatively young age group of miners where presbycusis was not a confounding factor. The aging population of miners, in the presence of high prevalence of TB and HIV, raises more implications for audiological and otological clinical service delivery in this population. This study therefore, raises important implications about hearing conservation programs for miners with a history of TB in this context. Adherence to the use of personal hearing protection devices within a carefully run ototoxicity/noise monitoring programmer, as well as use of ototoxic-protective/chemo-protective agents in the miners who are on TB medication, are important considerations, particularly since use of personal hearing protection devices cannot be guaranteed.

## Conflicts of interest

The author declares no conflicts of interest.
